# Adherence to Guidelines for the Administration of Intraoperative Antibiotics in a Nationwide US Sample

**DOI:** 10.1001/jamanetworkopen.2021.37296

**Published:** 2021-12-14

**Authors:** Amit Bardia, Miriam M. Treggiari, George Michel, Feng Dai, Mayanka Tickoo, Mabel Wai, Kevin Schuster, Michael Mathis, Nirav Shah, Sachin Kheterpal, Robert B. Schonberger

**Affiliations:** 1 Department of Anesthesiology, Yale School of Medicine, New Haven, Connecticut; 2 Yale Center for Analytical Sciences, New Haven, Connecticut; 3 Department of Medicine, Yale School of Medicine, New Haven, Connecticut; 4 Department of Pharmacy, Yale New Haven Hospital, New Haven, Connecticut; 5 Department of Surgery, Yale School of Medicine, New Haven, Connecticut; 6 Department of Anesthesiology, University of Michigan School of Medicine, Ann Arbor

## Abstract

**Importance:**

Despite widespread adherence to Surgical Care Improvement Project antibiotic measures, prevention of surgical site infections (SSIs) remains a clinical challenge. Several components of perioperative antibiotic prophylaxis guidelines are incompletely monitored and reported within the Surgical Care Improvement Project program.

**Objectives:**

To describe adherence to each component of perioperative antibiotic prophylaxis guidelines in regard to procedure-specific antibiotic choice, weight-adjusted dosing, and timing of first and subsequent administrations in a nationwide, multicenter cohort of patients undergoing noncardiac surgery.

**Design, Setting, and Participants:**

This cohort study included adult patients undergoing general, urological, orthopedic, and gynecological surgical procedures involving skin incision between January 1, 2014, and December 31, 2018, across 31 academic and community hospitals identified within the Multicenter Perioperative Outcomes Group registry. Data were analyzed between April 2 and April 21, 2021.

**Main Outcomes and Measures:**

The primary end point was overall adherence to Infectious Diseases Society of America guidelines, including (1) appropriateness of antibiotic choice, (2) weight-based dose adjustment, (3) timing of administration with respect to surgical incision, and (4) timing of redosing when indicated. Data were analyzed using mixed-effects regression to investigate patient, clinician, and institutional factors associated with guideline adherence.

**Results:**

In the final cohort of 414 851 encounters across 31 institutions, 51.8% of patients were women, the mean (SD) age was 57.5 (15.7) years, 1.2% of patients were of Hispanic ethnicity, and 10.2% were Black. In this cohort, 148 804 encounters (35.9%) did not adhere to guidelines: 19.7% for antibiotic choice, 17.1% for weight-adjusted dosing, 0.6% for timing of first dose, and 26.8% for redosing. In adjusted analyses, overall nonadherence was associated with emergency surgery (odds ratio [OR], 1.35; 95% CI, 1.29-1.41; *P* < .001), surgery requiring blood transfusions (OR, 1.30; 95% CI, 1.25-1.36; *P* < .001), off-hours procedures (OR, 1.08; 95% CI, 1.04-1.13; *P* < .001), and procedures staffed by a certified registered nurse anesthetist (OR, 1.14; 95% CI, 1.11-1.17; *P* < .001). Overall adherence to guidelines for antibiotic administration improved over the study period from 53.1% (95% CI, 52.7%-53.5%) in 2014 to 70.2% (95% CI, 69.8%-70.6%) in 2018 (*P* < .001).

**Conclusions and Relevance:**

In this cohort study, although adherence to perioperative antibiotic administration guidelines improved over the study period, more than one-third of surgical encounters remained discordant with Infectious Diseases Society of America recommendations. Future quality improvement efforts targeting gaps in practice in relation to guidelines may lead to improved adherence and possibly decreased SSIs.

## Key Points

**Question:**

What are the patterns of intraoperative antibiotic administration across US hospitals with respect to Infectious Diseases Society of America’s guidelines?

**Findings:**

In this multicenter cohort study comprising 414 851 surgical encounters across 31 institutions, more than one-third of the encounters did not adhere to guidelines. With the exception of timing of antibiotics, all measures of antibiotic administration (choice, dosing, and redosing) had marked nonadherence.

**Meaning:**

These findings suggest that considerable nonadherence to intraoperative antibiotic administration best practices persists, which may be a contributory factor to stagnant rates of surgical site infections.

## Teaser

This multicenter cohort study examines the patterns of adherence to the Infectious Disease Society of America’s guidelines for intraoperative antibiotic administration in US hospitals.

## Introduction

Surgical site infections (SSIs) are currently the leading cause of health care–related infections and unplanned hospital readmissions among surgical patients.^1,2,3,4,5^ Surgical site infections affect about 125 000 surgical cases annually, accounting for nearly 1 million excess hospital days and approximately $1.6 billion in annual incremental health care costs.^6^ Reduction of SSIs continues to be a major priority area in health care improvement because these events take a substantial toll on public health and health care resources.^7^ It is estimated that half of SSIs are preventable, and efforts directed at the prevention of SSIs have been declared a priority objective by the US Department of Health and Human Services.^8,9^ However, over the past several years, SSI rates have remained stagnant despite the introduction of specific measures and surveillance programs geared toward SSI reduction.^10,11,12^

The etiology of SSIs is multifactorial, and although not all risk factors are modifiable, the inappropriate administration of perioperative antibiotics has the potential to contribute to the problem. Importantly, antibiotic management represents a potentially modifiable risk factor. The critical role of appropriate perioperative antibiotics in preventing SSIs has been well established^3,13,14,15,16^ and has been among the key initiatives of the Surgical Care Improvement Project (SCIP).^17^ The SCIP guidelines primarily focus on timing of antibiotics prior to surgery and antibiotic choice for a subset of selected surgical procedures. Whereas the SCIP antibiotic metrics have been a major focus of quality improvement efforts, little information has been reported regarding adherence to additional recommendations contained in the more extensive guidelines endorsed by the American Society of Health-System Pharmacists, the Infectious Diseases Society of America (IDSA), the Surgical Infection Society, and the Society for Healthcare Epidemiology of America.^18^ The salient features of these guidelines for perioperative antibiotic prophylaxis include choice of antibiotics tailored to type of surgery, weight-based antibiotic dose adjustment, completion of antibiotic administration prior to skin incision, and intraoperative redosing at specific intervals. The primary objective of this study was to describe the prevalence of guideline adherent practices for antibiotic prophylaxis during surgery among centers participating in the Multicenter Perioperative Outcomes Group (MPOG) consortium, a large research and quality improvement consortium based at the University of Michigan.

## Methods

### Data Source

This multicenter observational study was approved by the Yale University institutional review board in collaboration with the MPOG,^19,20^ with a waiver of informed consent for the use of deidentified data. The MPOG database includes anesthetic encounters from a variety of academic and community hospitals across 21 states.^21^ Methods for data collection, validation, mapping to universal concepts interoperable across sites, and secure transfer to a coordinating center are previously described.^22^ Data validation includes both automated data quality monitoring by the coordinating center as well as case-by-case validation of a monthly sample of data by investigators at each contributing institution. The MPOG Perioperative Clinical Research Committee approved the analytic plan that was published prior to data analysis.^23^ The study was conducted in accordance with the Reporting of Studies Conducted Using Observational Routinely Collected Health Data (RECORD) statement, an extension of the Strengthening the Reporting of Observational Studies in Epidemiology (STROBE) reporting guideline.^24,25^

### Study Population

Intraoperative records of patients aged 18 years or older who underwent general, orthopedic, gynecological, and urological surgical procedures involving a skin incision between January 1, 2014, and December 31, 2018, were eligible for inclusion. We excluded patients with the following characteristics: ongoing preoperative antibiotic therapy, missing intraoperative antibiotic documentation, missing American Society of Anesthesiologists score, or missing weight (eFigure 1 in the Supplement). Each surgical event was treated as a unique patient encounter.

### End Points

The primary end point of this study was to determine the proportion of adherence to the recommendations for intraoperative antibiotic administration as stated in the IDSA, Surgical Infection Society, American Society of Health-System Pharmacists, and Society for Healthcare Epidemiology guidelines.^18^ We defined appropriate perioperative antibiotic prophylaxis based on adherence to the following metrics: (1) appropriateness of procedure-specific antibiotic choice, (2) appropriateness of weight-based dose adjustment, (3) timing of antibiotic administration prior to surgical incision, and (4) timing of intraoperative redosing.

#### Appropriateness of Antibiotic Choice

Appropriateness of antibiotic choice was determined based on procedure type identified using *Current Procedure Terminology *(*CPT*) codes (eAppendix 2 in the Supplement). Because patient (eg, drug allergies) and hospital (eg, pharmacy availability) characteristics may necessitate the use of second-line agents, we considered the antibiotic choice as appropriate if either first- or second-line antibiotics from the guidelines’ listed procedural category were documented. In cases in which more than 1 antibiotic was administered, at least 1 antibiotic or a combination of antibiotics needed to be consistent with first- or second-line antibiotic recommendations.

#### Accuracy of Weight-Based Dose Adjustment

Adherence to the recommendation for weight-based dose adjustment was considered successful if an appropriate weight-adjusted antibiotic dose was administered. For antibiotic dosing in which guidelines state a milligram per kilogram calculation (eg, vancomycin), doses that were at least 90% of the correct dose were considered as guideline adherent.

#### Timing of Antibiotic Administration Prior to Surgical Incision

For boluses, we used the documented time of antibiotic administration, and for infusions, we used the start of infusion administration as the qualifying administration time prior to incision. Adherence to this recommendation was considered successful if the antibiotic administration was documented within the time window established by the guidelines.

#### Timing of Intraoperative Redosing

Instances qualifying for redosing of antibiotics were identified when the duration of the surgery was longer than the minimum interval(s) for which redosing was recommended per guidelines. Adherence to this recommendation was considered successful if all required subsequent antibiotic administrations were documented prior to the end of surgery. Failure of any expected redosing event was adjudicated as failure of redosing.

### Covariates

For each surgical encounter, age at the time of surgery, gender, race, ethnicity, body mass index, and American Society of Anesthesiologists physical status classification were queried. Procedural characteristics that were examined included hospital setting (teaching vs community hospital based on medical school affiliation), year when the surgery was performed, surgical specialty of the primary surgeon, anesthesist type (resident, certified registered nurse anesthetist, or solo anesthesiologist), urgency of surgery (emergent vs nonemergent), regular vs off-hours (5:00 pm to 6:30 am), surgery start time, use of blood products, vasopressor infusions, and duration of surgery.

### Statistical Analysis

Data were analyzed between April 2 and April 21, 2021. Categorical variables were described using frequency distributions and proportions. Medians and IQRs as well as means and standard deviations were used to summarize continuous variables. We used descriptive statistics to quantify the frequencies and percentages of adherence with 95% CIs for overall and institution-specific antibiotic guideline adherence. Overall adherence required all 4 relevant domains of antibiotic adherence to have been satisfied. Each metric was then individually appraised to examine the variation in adherence for the metric of interest. The χ^2^ and *t* test or Mann-Whitney U test were used to compare distributions as appropriate. Unadjusted analyses are presented using descriptive statistics by institution, with caterpillar plots to display the proportion of guideline adherence. To investigate associations between patient-, clinician-, and institution-level factors and overall adherence, we used logistic mixed effects regression models. Institutions were treated as having a random intercept to account for clustering of patients within each institution. All 2-sided *P* < .05 were considered significant.

## Results

### Baseline Characteristics

In the final cohort of 414 851 encounters across 31 institutions, 51.8% of participants were women, 48.2% were men, and the mean (SD) age was 57.5 (15.7) years. Overall, 1.2% of participants were of Hispanic ethnicity; 10.2% were Black, 71.2% were White, 14.2% were of unknown race, and 4.4% were of other race (including American Indian or Alaska Native, Asian, and Native Hawaiian or other Pacific Islander). In this cohort, 148 804 encounters (35.9%) were found to not be guideline adherent. Table 1 summarizes the surgical encounter characteristics stratified by overall guideline adherence. Clinically unimportant differences between guideline-adherent and nonadherent cases were observed for mean (SD) age (guideline-adherent: 57.6 [15.7] years vs guideline nonadherent: 57.4 [15.8] years), sex (female nonadherent: 35.2% vs male nonadherent: 36.5%) and mean (SD) body mass index (adherent: 29.4 [6.8] vs nonadherent: 29.0 [7.3]). Similarly, the median duration of surgery was comparable in the 2 groups (median adherent: 183.0 minutes [IQR, 130.0-253.0 minutes] vs nonadherent: 180.0 minutes [IQR, 115.0-281.0 minutes]). The large majority of cases were listed as nonemergency (96.9%) and were performed between 6:30 am and 5:00 pm (96.1%).

**Table 1.** Baseline Demographic and Clinical Characteristics Stratified by Overall Adherence to Antibiotic Administration Recommendations per the Infectious Diseases Society of America Guidelines

Variable	No. (%)		
	All patients (N = 414 851)	Overall guideline adherent (N = 266 047)	Overall guideline nonadherent (N = 148 804)
Age, mean (SD), y	57.5 (15.7)	57.6 (15.7)	57.4 (15.8)
Female	214 960	139 203 (64.8)	75 757 (35.2)
Male	199 891	126 844 (63.5)	73 047 (36.5)
BMI, mean (SD)	29.2 (7.0)	29.4 (6.8)	29.0 (7.3)
Hispanic ethnicity	4872	2861 (58.7)	2011 (41.3)
Race			
Black	42 416	27 714 (65.3)	14 702 (34.7)
White	295 220	186 333 (63.1)	108 887 (36.9)
Unknown	59 015	39 799 (67.4)	19 216 (32.6)
Other^a^	18 200	12 201 (67.0)	5999 (33.0)
Surgical specialty			
General surgery	186 711	99 985 (53.6)	86 726 (46.4)
Gynecology	41 832	30 340 (72.5)	11 492 (27.5)
Orthopedics	120 015	97 224 (81.0)	22 791 (19.0)
Urology	66 293	38 498 (58.1)	27 795 (41.9)
Duration of surgery, median (IQR), min	182.0 (125.0-261.0)	183.0 (130.0-253.0)	180.0 (115.0-281.0)
ASA class^b^			
1	24 736	16 369 (66.2)	8367 (33.8)
2	180 336	120 594 (66.9)	59 742 (33.1)
3	193 926	119 783 (61.8)	74 143 (38.2)
4	15 514	9117 (58.8)	6397 (41.2)
5	339	184 (54.3)	155 (45.7)
Blood products given	13 547	7620 (56.2)	5927 (43.8)
Vasopressor infusion use	74 094	52 979 (71.5)	21 115 (28.5)
Supervision			
CRNA	240 433	145 134 (60.4)	95 299 (39.6)
Combination^c^	28 833	17 711 (61.4)	11 122 (38.6)
Resident	105 243	75 358 (71.6)	29 885 (28.4)
Solo	40 342	27 844 (69.0)	12 498 (31.0)
Off-hours cases^d^	16 212	9691 (59.8)	6521 (40.2)
Year of surgery			
2014	63 053	33 458 (53.1)	29 595 (46.9)
2015	86 761	52 846 (60.9)	33 915 (39.1)
2016	100 325	65 620 (65.4)	34 705 (34.6)
2017	117 377	80 907 (68.9)	36 470 (31.1)
2018	47 335	33 216 (70.2)	14 119 (29.8)
Emergency case			
Emergency	12 950	7444 (57.5)	5506 (42.5)
Nonemergency	401 901	258 603 (64.3)	143 298 (35.7)

Abbreviations: ASA, American Society of Anesthesiologists; BMI, body mass index (calculated as weight in kilograms divided by height in meters squared); CRNA, certified registered nurse anesthetist.

^a^ Other includes American Indian or Alaska Native, Asian, and Native Hawaiian or other Pacific Islander.

^b^ The purpose of the ASA system is to assess and communicate a patient’s preanesthesia medical comorbidities, with 1 indicating a healthy patient and 5 indicating a patient who is not expected to survive without the surgery.

^c^ Cases involving 2 of the following: solo anesthesiologist, CRNA with anesthesiologist, and resident with anesthesiologist.

^d^ Cases starting between 5:00 pm and 6:30 am.

Regarding adherence across the 4 individual metrics, adherence to timing of first dose administration—the one metric captured by SCIP public reporting—was 99.4% (95% CI, 99.4%-99.5%) (eFigure 2, eTable 2 in the Supplement). Adherence to the 3 non-SCIP reported metrics was as follows: adherence to redosing guidelines, 73.2% (95% CI, 72.9%-73.5%); adherence to weight-adjusted dosing, 82.9% (95% CI, 82.8%-83.0%); and adherence to procedure-specific drug, 80.4% (95% CI, 80.2%-80.5%) (Table 2).

**Table 2.** Antibiotic Guideline Adherence for Each Individual Metric, Stratified by Overall Adherence

Metric	All patients, No.	No. (%)	
		Guideline adherent	Guideline nonadherent
Overall	414 851	266 047 (64.1)	148 804 (35.9)
Choice of antibiotic	414 851	333 338 (80.4)	81 513 (19.7)
Weight-based dose adjustment	414 851	343 835 (82.9)	71 016 (17.1)
Time of first dose	414 851	412 523 (99.4)	2328 (0.6)
Time of redosing^a^	68 776	50 334 (73.2)	18 442 (26.8)

^a^ Only surgical cases with a duration of surgery greater than the antibiotic redosing interval were included to calculate adherence to redosing guidance.

Among the individual antibiotics, vancomycin was most frequently underdosed, with 50.5% of vancomycin encounters receiving less than 90% of the recommended weight-adjusted dose. The proportion of surgical cases with nonadherence for the 2 most commonly used antibiotics that qualified for guideline based redosing—cefazolin and cefoxitin—was 20.9% and 74.2%, respectively.

### Factors Associated With Nonadherence to Antibiotic Administration Guidelines

In adjusted analyses (Table 3), we found that emergency surgery (OR, 1.35, 95% CI, 1.29-1.41; *P* < .001), procedures starting during off-hour shifts (ie, not between 6:30 am and 5:00 pm) (OR, 1.08; 95% CI, 1.04-1.13; *P* < .001), and surgery requiring blood transfusions (OR, 1.30; 95% CI, 1.25-1.36; *P* < .001) were associated with guideline nonadherence. Among surgical specialties, orthopedic surgery (OR, 0.26; 95% CI, 0.25-0.26), gynecology (OR, 0.38; 95% CI, 0.37-0.39), and urology (OR, 0.74; 95% CI, 0.73-0.76) were associated with higher guideline adherence compared with general surgery. Relative to solo anesthesiologists, cases performed with residents had lower odds of nonadherence (OR, 0.90; 95% CI, 0.87-0.92), whereas cases performed with certified registered nurse anesthetists (OR, 1.14; 95% CI, 1.11-1.17; *P* < .001) had higher odds of guideline nonadherence. The unadjusted and adjusted analyses of factors associated with guideline nonadherence for the 4 individual metrics are shown in eTables 1 through 8 in the Supplement.

**Table 3.** Multivariable Analysis Estimating the Association of Patient-Level Factors Associated With Overall Guideline-Nonadherent Antibiotic Administration

Variable	Odds ratio (95% CI)	*P* value
Age	0.99 (0.99-1.00)	.002
Gender, male vs female	0.95 (0.93-0.96)	<.001
BMI	0.99 (0.99-0.99)	<.001
Ethnicity, Hispanic vs Non-Hispanic	1.01 (0.94-1.07)	.84
Race		
Black	0.93 (0.91-0.96)	<.001
White	1 [Reference]	NA
Other^a^	0.95 (0.91-0.98)	.002
Unknown	1.02 (0.99-1.04)	.20
Surgical specialty		
Gynecology	0.38 (0.37-0.39)	<.001
Orthopedics	0.26 (0.25-0.26)	<.001
Urology	0.74 (0.73-0.76)	<.001
General surgery	1 [Reference]	NA
Duration of surgery, min	1.01 (1.01-1.01)	<.001
ASA class^b^		
2	0.87 (0.85-0.90)	<.001
3	0.88 (0.85-0.91)	<.001
4	0.92 (0.87-0.96)	.004
5	0.80 (0.63-1.02)	.07
1	1 [Reference]	NA
Blood products given, yes vs no	1.30 (1.25-1.36)	<.001
Vasopressor use, yes vs no	0.91 (0.89-0.93)	<.001
Supervision		
CRNA	1.14 (1.11-1.17)	<.001
Combination^c^	1.09 (1.05-1.13)	<.001
Resident	0.90 (0.87-0.92)	<.001
Solo	1 [Reference]	NA
Off-hours cases (starting between 5:00 pm and 6:30 am), yes vs no	1.08 (1.04-1.13)	<.001
Year of surgery		
2015	0.65 (0.64-0.67)	<.001
2016	0.56 (0.55-0.58)	<.001
2017	0.54 (0.52-0.55)	<.001
2018	0.51 (0.50-0.53)	<.001
2014	1 [Reference]	NA
Emergency case, yes vs no	1.35 (1.29-1.41)	<.001

Abbreviations: ASA, American Society of Anesthesiologists; BMI, body mass index (calculated as weight in kilograms divided by height in meters squared); CRNA, certified registered nurse anesthetist; NA, not applicable.

^a^ Other includes American Indian or Alaska Native, Asian, and Native Hawaiian or other Pacific Islander.

^b^ The purpose of the ASA system is to assess and communicate a patient’s preanesthesia medical comorbidities, with 1 indicating a healthy patient and 5 indicating a patient who is not expected to survive without the surgery.

^c^ Cases involving 2 of the following: solo anesthesiologist, CRNA with anesthesiologist, and resident with anesthesiologist.

### Trends Over Time and Institutional Variation in Adherence to Antibiotic Administration Guidelines

**Figure 1.**
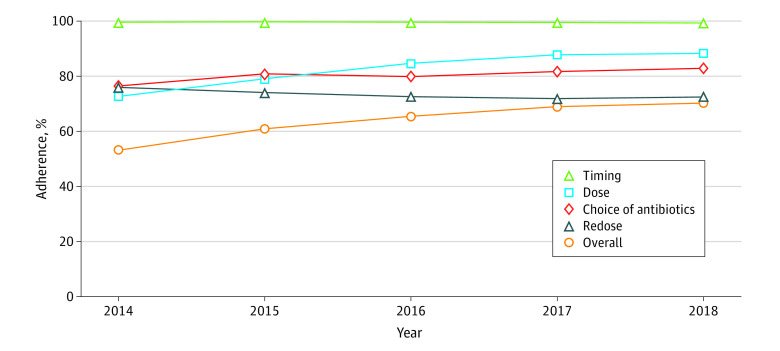
Temporal Trends in Adherence to Perioperative Antibiotic Administration Guidelines From 2014 to 2018 The overall adherence to guideline-based antibiotic administration was noted to improve over time. The overall rates of adherence in the 4 domains were 80.4% for choice, 99.4% for timing, 82.9% for dosing, and 73.2% for redosing.

**Figure 2.**
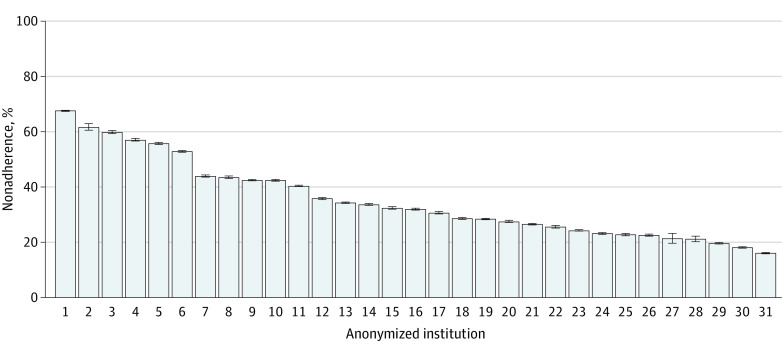
Rate of Nonadherence by Institution Center-specific adjusted antibiotic nonadherence rates across the 31 centers are depicted for the study period (January 1, 2014, to December 31, 2018). The adjusted noncompliance ranged from 16.1% to 67.6%. Error bars indicate 95% CIs.

The change by year in guideline adherence with respect to each metric and their composite is shown in Figure 1. The overall adherence to guideline-based antibiotic administration improved from 53.1% (95% CI, 52.7%-53.5%) in 2014 to 70.2% (95% CI, 69.8%-70.6%) in 2018 (*P* < .001). A post hoc test for trend further demonstrated a positive association of antibiotic adherence by center from 2014 to 2018 (β = 0.14, *P* < .001). Center-specific adjusted antibiotic nonadherence across the 31 centers ranged from a point estimate (SE) of 16.11% (0.05%) to 67.64% (0.05%) (Figure 2).

In a preliminary post hoc analysis (eAppendix 1 in the Supplement) comparing antibiotic nonadherence with SSIs after colon operations and abdominal hysterectomy extracted from a publicly available hospital compare registry, we found no association between hospital performance tertile and IDSA adherence rate in colon surgery group (OR, 0.97; 95% CI, 0.89-1.04; *P* = .36). The association was also not statistically significant in the abdominal hysterectomy group (OR, 0.95; 95% CI, 0.84-1.08; *P* = .46). It should be noted that the above analysis to approach the question of potential associations among IDSA metrics and SSI rates does not include accounting for case-mix or numerous other important confounders.

## Discussion

In this cohort of 414 851 noncardiac surgical encounters across 31 institutions, we observed that 148 804 encounters (35.9%) were nonadherent to IDSA guidelines for perioperative antibiotic administration. With the exception of the SCIP metric of antibiotic timing, substantial nonadherence to the guidelines for perioperative antibiotic administration was found across all examined domains, including appropriate procedure-specific antibiotic choice, weight-adjusted dosing, and timely redosing of antibiotics. Factors associated with overall guideline nonadherence were emergency cases, those requiring blood transfusions, and those performed during off hours. Additionally, although the overall adherence to guidelines improved across the study years from 53.1% in 2014 to 70.2% in 2018, it still remained suboptimal, with substantial room for improvement across the 3 domains not included in SCIP. Although further studies are needed to determine the association and relative importance of the various components of the guidelines and SSI outcomes, previous work reported associations between adequate presurgical antibiotic administration and lower rates of SSIs.^26,27,28^

Emergency surgical procedures and increased blood transfusions have been reported to be associated with a higher rate of SSIs.^29,30,31^ Interestingly, the findings of our study show these factors to also be associated with antibiotic nonadherence. Although maximal prevention of SSIs is a multifactorial challenge, improved adherence to IDSA guidelines may be one critical step toward decreasing overall rates of SSIs.

In order to improve surgical outcomes, a number of quality improvement measures have been undertaken to promote guideline adherence practices with variable success.^32,33,34,35,36,37,38^ Owing to near-universal adherence to SCIP metrics, some practitioners may incorrectly consider perioperative antibiotic prophylaxis to be a solved problem. To the contrary, the present study identifies key opportunities for further improvement regarding best practices for perioperative antibiotic administration. These findings are broadly consistent with those of other studies evaluating guideline-based intraoperative antibiotics administration that have also reported low to modest adherence depending on the type of surgery and the study population investigated.^28,39,40,41^

In terms of specific antibiotics that may benefit from focused quality improvement initiatives, we identified certain medications that may warrant closer attention when administered. Our findings indicate that 50.5% of the patients administered vancomycin received a dose at least 10% lower than guidelines would dictate. Generally, vancomycin is the preferred antibiotic for patients with methicillin-resistant *Staphylococcus aureus* colonization,^18^ frequently seen in high-risk patients in health care settings. Using lower than recommended vancomycin doses may be especially deleterious owing to the potentially increased risk of SSIs in these patients.^42,43^ Moreover, nonadherence to guideline-based vancomycin administration has been linked to an increased rate of SSIs.^27,44^ Targeting efforts at optimizing vancomycin administration, especially related to its dose, may thus have an impact on reducing SSIs.

It is worth emphasizing that we found very high adherence to guidelines with respect to timing of initial antibiotic administration (99.4%) as compared with other metrics. Intense attention has been given to appropriate timing of antibiotics in the context of the SCIP initiative, which likely explains the excellent performance in this metric across institutions. The success in adherence to SCIP suggests that implementation of similar initiatives targeting a more comprehensive set of metrics relevant to appropriate antibiotic administration may similarly improve adherence.

### Limitations

Despite its merits, our study has some limitations. First, this retrospective observational study has the pitfalls associated with this type of study design; however, the MPOG data have been extracted with several robust steps in place to enhance reliability and have been used in a variety of high-quality observational studies. Second, the link between nonadherent practices and increased rates of SSIs remains to be determined. In our post hoc exploratory analysis, we found no association between hospital SSI performance tertile and antibiotic adherence for colon operations and abdominal hysterectomy. However, as mentioned above, strong evidence from a number of prior studies have shaped the current antibiotic prophylaxis guidelines, and substantial evidence exists to show that nonstandard antibiotic administration practices are associated with increased SSIs. Third, we excluded patients who did not have any antibiotic documented in the anesthesia record. This exclusion was planned by design to avoid making assumptions about the reasons why documentation was missing. Finally, our procedure-specific antibiotic assessments were based on the primary *CPT* of the surgical procedure. It is possible that additional *CPT* codes not included in the primary *CPT* would have led some apparently guideline-adherent surgical procedures to in fact be nonadherent. Any such errors would have led to greater levels of nonadherence than what we reported here. Moreover, we are unable to comment on inappropriate extension of antibiotics after surgery because the current registry does not record antibiotic administration data beyond the operating room.

A further limitation regards the issue of attribution. We did not attempt to elucidate the causes of nonadherence nor to attribute it to specific health care professionals. Although the IDSA guidelines were chosen as a reference for this study, it is possible that patients received antibiotics according to a subspecialty-specific guideline that differs from the standard IDSA guideline. However, these guidelines are based on similar evidence, share similar features, and are, we believe, the most widely used. Similarly, it is possible that health care professionals who were nonadherent to the guidelines were following institutional protocols that may not be reflective of the most current IDSA standards. Further exploration of institutional variation in antibiotic protocols would help clarify the issue of attribution and thus direct future quality improvement initiatives. Additionally, the underlying causes of these trends in antibiotic care cannot be determined from our data. Both individual- and hospital-based factors may be driving this change. Despite these limitations, we believe our study provides valuable data on large-scale patterns related to antibiotic nonadherence for various surgical procedures across a number of institutions in the US.

## Conclusions

Although adherence to perioperative antibiotic administration guidelines has improved over time, the findings of this cohort study suggest that substantial nonadherence persists. Our study highlights opportunities for intervention and suggests that a more comprehensive approach to evaluate guideline adherence beyond SCIP for the optimal management of perioperative antibiotic prophylaxis is needed. Future quality improvement efforts directed at improving antibiotic guideline adherence may lead to a decrease in SSIs and improved surgical outcomes. The effect of this nonadherence on SSIs needs to be further explored in future studies.

## Notes

Supplement. **eFigure 1.** Patient Flowchart **eFigure 2.** Adherence by Each Metric **eTable 1.** Baseline Demographic and Clinical Characteristics Stratified by Antibiotic Usage With Respect to Timing of Antibiotics at the Start of a Surgery per the IDSA Guidelines in the Study Population **eTable 2.** Multivariable Analysis Evaluating the Association of Demographic and Perioperative Factors Associated With Guideline Nonadherent Antibiotic Usage With Respect to Timing Of Antibiotics at the Start of a Surgery **eTable 3.** Baseline Demographic and Clinical Characteristics Stratified by Antibiotic Usage With Respect to Dosing of Antibiotics per the IDSA Guidelines in the Study Population **eTable 4.** Multivariable Analysis Evaluating the Association of Demographic and Perioperative Factors Associated With Guideline Nonadherent Antibiotic Usage With Respect to Dosing of Antibiotics **eTable 5.** Baseline Demographic and Clinical Characteristics Stratified by Antibiotic Usage With Respect to Choice of Antibiotics per the IDSA Guidelines in the Study Population **eTable 6.** Multivariable Analysis Evaluating the Association of Demographic and Perioperative Factors Associated With Guideline Nonadherent Antibiotic Usage With Respect to the Choice of Antibiotics for a Surgery **eTable 7.** Baseline Demographic and Clinical Characteristics Stratified by Antibiotic Usage With Respect to Redosing of Antibiotics per the IDSA Guidelines in the Study Population **eTable 8.** Multivariable Regression Analysis Evaluating the Association of Demographic and Perioperative Factors Associated With Guideline Nonadherent Antibiotic Usage With Respect to Redosing of Antibiotics During a Surgery **eAppendix 1.** Post Hoc Analysis Evaluating Association of SSIs After Colon Surgeries and Abdominal Hysterectomy with Antibiotic Adherence at a Hospital Level **eAppendix 2.** Current Procedure Terminology (CPT) Codes by Surgical Specialty
