# Supplementary material for: Adherence to Guidelines for the Administration of Intraoperative Antibiotics in a Nationwide US Sample
Source: JAMA Netw Open. 2021 Dec 14;4(12):e2137296. doi: 10.1001/jamanetworkopen.2021.37296 (PMC8672234; doi:10.1001/jamanetworkopen.2021.37296)
Supplement: Supplement. — eFigure 1. Patient Flowchart eFigure 2. Adherence by Each Metric eTable 1. Baseline Demographic and Clinical Characteristics Stratified by Antibiotic Usage With Respect to Timing of Antibiotics at the Start of a Surgery per the IDSA Guidelines in the Study Population eTable 2. Multivariable Analysis Evaluating the Association of Demographic and Perioperative Factors Associated With Guideline Nonadherent Antibiotic Usage With Respect to Timing Of Antibiotics at the Start of a Surgery eTable 3. Baseline Demographic and Clinical Characteristics Stratified by Antibiotic Usage With Respect to Dosing of Antibiotics per the IDSA Guidelines in the Study Population eTable 4. Multivariable Analysis Evaluating the Association of Demographic and Perioperative Factors Associated With Guideline Nonadherent Antibiotic Usage With Respect to Dosing of Antibiotics eTable 5. Baseline Demographic and Clinical Characteristics Stratified by Antibiotic Usage With Respect to Choice of Antibiotics per the IDSA Guidelines in the Study Population eTable 6. Multivariable Analysis Evaluating the Association of Demographic and Perioperative Factors Associated With Guideline Nonadherent Antibiotic Usage With Respect to the Choice of Antibiotics for a Surgery eTable 7. Baseline Demographic and Clinical Characteristics Stratified by Antibiotic Usage With Respect to Redosing of Antibiotics per the IDSA Guidelines in the Study Population eTable 8. Multivariable Regression Analysis Evaluating the Association of Demographic and Perioperative Factors Associated With Guideline Nonadherent Antibiotic Usage With Respect to Redosing of Antibiotics During a Surgery eAppendix 1. Post Hoc Analysis Evaluating Association of SSIs After Colon Surgeries and Abdominal Hysterectomy with Antibiotic Adherence at a Hospital Level eAppendix 2. Current Procedure Terminology (CPT) Codes by Surgical Specialty [file jamanetwopen-e2137296-s001.pdf]

Supplemental Online Content

Bardia A, Treggiari MM, Michel G, et al. Adherence to guidelines for the administration of intraoperative antibiotics in a nationwide US sample. *JAMA Netw Open.* 2021;4(12):e2137296. doi:10.1001/jamanetworkopen.2021.37296

eFigure 1. Patient Flowchart

eFigure 2. Adherence by Each Metric

eTable 1. Baseline Demographic and Clinical Characteristics Stratified by Antibiotic Usage With Respect to Timing of Antibiotics at the Start of a Surgery per the IDSA Guidelines in the Study Population

eTable 2. Multivariable Analysis Evaluating the Association of Demographic and Perioperative Factors Associated With Guideline Nonadherent Antibiotic Usage With Respect to Timing Of Antibiotics at the Start of a Surgery

eTable 3. Baseline Demographic and Clinical Characteristics Stratified by Antibiotic Usage With Respect to Dosing of Antibiotics per the IDSA Guidelines in the Study Population

eTable 4. Multivariable Analysis Evaluating the Association of Demographic and Perioperative Factors Associated With Guideline Nonadherent Antibiotic Usage With Respect to Dosing of Antibiotics

eTable 5. Baseline Demographic and Clinical Characteristics stratified by Antibiotic Usage With Respect to Choice of Antibiotics per the IDSA Guidelines in the Study Population

eTable 6. Multivariable Analysis Evaluating the Association of Demographic and Perioperative Factors Associated With Guideline Nonadherent Antibiotic Usage With Respect to the Choice of Antibiotics for a Surgery

eTable 7. Baseline Demographic and Clinical Characteristics Stratified by Antibiotic Usage With Respect to Redosing of Antibiotics per the IDSA Guidelines in the Study Population

eTable 8. Multivariable Regression Analysis Evaluating the Association of Demographic and Perioperative Factors Associated With Guideline Nonadherent Antibiotic Usage With Respect to Redosing of Antibiotics During a Surgery

eAppendix 1. Post Hoc Analysis Evaluating Association of SSIs After Colon Surgeries and Abdominal Hysterectomy with Antibiotic Adherence at a Hospital Level

eAppendix 2. Current Procedure Terminology (CPT) Codes by Surgical Specialty

This supplemental material has been provided by the authors to give readers additional information about their work.

© 2021 Bardia A et al. *JAMA Network Open.*

eFigure 1. Patient Flowchart. Adult patients who underwent surgery from 01/01/2014 to 12/31/2018 were assessed using the MPOG database. After exclusions a total of 414,851 cases were included for analysis.

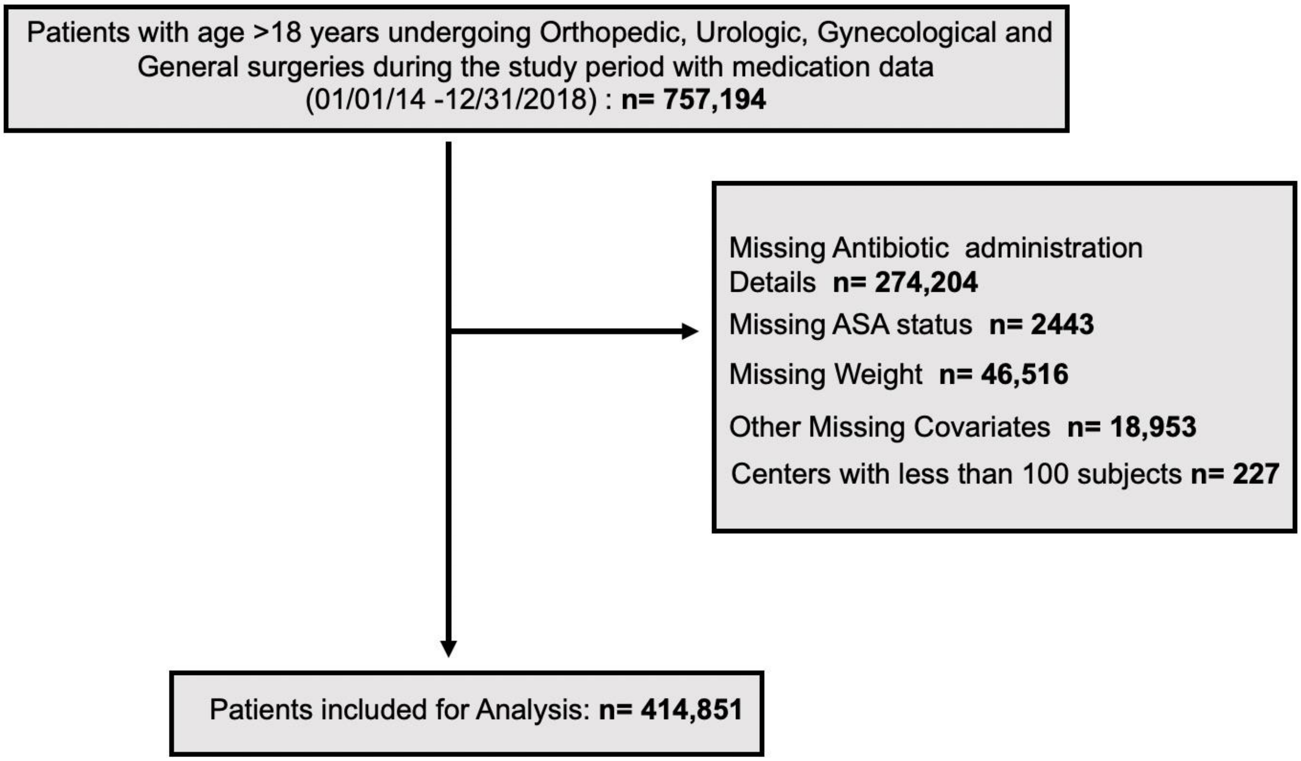

© 2021 Bardia A et al. *JAMA Network Open.*

eFigure 2. Adherence by Each Metric. Venn diagram depicting overall Antibiotic adherence for each individual adherence metric in the study cohort across 31 institutions, stratified by overall adherence. (Not drawn to scale)

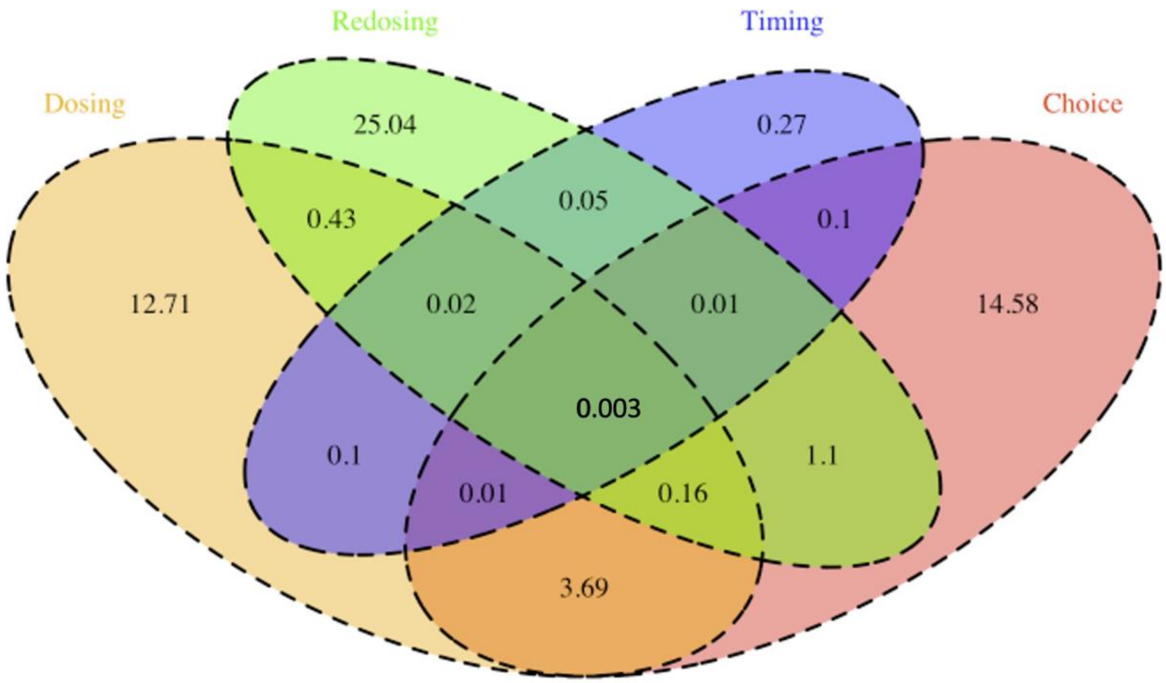

© 2021 Bardia A et al. *JAMA Network Open.*

eTable 1. Baseline Demographic and Clinical Characteristics Stratified by Antibiotic Usage With Respect to Timing of Antibiotics at the Start of a Surgery per the IDSA Guidelines in the Study Population.

Variable	Overall (N = 414851)	Guideline adherent timing of antibiotic (N = 412523)	Guideline non-adherent timing of antibiotic (N = 2328)	p-Value
**Age,** Mean (SD)	57.5 (15.7)	57.5 (15.7)	58.4 (15.6)	0.007
**Female,** n (%)	214,960 (51.8%)	213,771 (99.4%)	1189 (0.6%)	0.47
**BMI,** Mean (SD)	29.2 (7.0)	29.2 (7.0)	29.3 (7.5)	0.46
**Hispanic Ethnicity**	4872 (1.2%)	4833 (99.2%)	39 (0.8%)	0.024
**Race**				<0.001
Black	42,416 (10.2%)	42,112 (99.3%)	304 (0.7%)	
Others[1]	18,200 (4.4%)	18,128 (99.6%)	72 (0.4%)	
Unknown	59,015 (14.2%)	58,699 (99.5%)	316 (0.5%)	
White	295,220 (71.2%)	293,584 (99.4%)	1636 (0.6%)	
**Surgical Specialty**				<0.001
General Surgery	186,711 (45.0%)	185,561 (99.4%)	1150 (0.6%)	
Gynecology	41,832 (10.1%)	41,698 (99.7%)	134 (0.3%)	
Orthopedics	120,015 (28.9%)	119,150 (99.3%)	865 (0.7%)	
Urology	66,293 (16.0%)	66,114 (99.7%)	179 (0.3%)	
**Duration of surgery** Median (IQR)	182.0 (125.0 – 261.0)	182.0 (124.0 – 260.0)	288.0 (213.0 – 393.0)	<0.001
**ASA class**				
1	24,736 (6.0%)	24,686 (99.8%)	50 (0.2%)	<0.001
2	180,336 (43.5%)	179,756 (99.7%)	580 (0.3%)	
3	193,926 (46.7%)	192,467 (99.2%)	1459 (0.8%)	
4	15,514 (3.7%)	15,286 (98.5%)	228 (1.5%)	
5	339 (0.1%)	328 (96.8%)	11 (3.2%)	
**Cases with blood products given,** n (%)	13,547 (3.3%)	13,273 (98.0%)	274 (2.0%)	<0.001
**Cases with Vasopressor infusion use,** n (%)	74,094 (17.9%)	73,426 (99.1%)	668 (0.9%)	<0.001
**Supervision**				<0.001
CRNA	240,433 (58.0%)	239,182 (99.5%)	1251 (0.5%)	
Combination[2]	28,833 (7.0%)	28,566 (99.1%)	267 (0.9%)	
Resident	105,243 (25.4%)	104,575 (99.4%)	668 (0.6%)	
Solo	40,342 (9.7%)	40,200 (99.6%)	142 (0.4%)	
**Off-hours Cases[3],** n(%)	16,212 (3.9%)	16,069 (99.1%)	143 (0.9%)	<0.001
**Year of Surgery**				<0.001
2014	63,053 (15.2%)	62,729 (99.5%)	324 (0.5%)	
2015	86,761 (20.9%)	86,371 (99.6%)	390 (0.4%)	
2016	100,325 (24.2%)	998,54 (99.5%)	471 (0.5%)	
2017	117,377 (28.3%)	116,618 (99.4%)	759 (0.6%)	
2018	47,335 (11.4%)	46,951 (99.2%)	384 (0.8%)	
**Emergency Cases**	12,950 (3.1%)	12,768 (98.6%)	182 (1.4%)	<0.001

© 2021 Bardia A et al. *JAMA Network Open.*

[1] Others: American Indian or Alaska Native, Asian, Native Hawaiian or Other Pacific Islander. [2] Combination: Cases involving 2 of the following: solo anesthesiologist, CRNA with anesthesiologist, resident with anesthesiologist. [3] Cases starting between 5:00PM and 6:30AM.

© 2021 Bardia A et al. *JAMA Network Open.*

eTable 2. Multivariable Analysis Evaluating the Association of Demographic and Perioperative Factors Associated With Guideline Nonadherent Antibiotic Usage With Respect to Timing Of Antibiotics at the Start of a Surgery.
(Non-adherent yes vs no)

Variable	Odds Ratio (95% CI)	p-Value
**Age**	0.917 (0.89 to 0.945)	<0.0001
**Gender, Male vs. female**	0.97 (0.89 to 1.06)	0.5474
**BMI**	0.995 (0.989 to 1.001)	0.0849
**Ethnicity, Hispanic vs. Non-Hispanic**	0.97 (0.70 to 1.34)	0.8341
**Race**		
Black	1.03 (0.90 to 1.18)	0.6727
Others[1]	0.77 (0.60 to 0.98)	0.0349
Unknown	1.13 (0.98 to 1.30)	0.0931
White	Ref	
**Surgical Specialty**		
Gynecology	0.64 (0.53 to 0.77)	<0.0001
Orthopedics	1.51(1.37 to 1.66)	<0.0001
Urology	0.42 (0.36 to 0.50)	<0.0001
General Surgery	Ref	
**Duration of surgery (min)**	1.048 (1.046 to 1.051)	<0.0001
**ASA class**		
2	1.26 (0.93 to 1.69)	0.1326
3	2.11 (1.57 to 2.85)	<0.0001
4	4.24 (3.05 to 5.89)	<0.0001
5	5.46 (2.68 to 11.16)	<0.0001
1	Ref	
**Blood products given, Yes vs. No**	0.92 (0.79 to 1.07)	0.2888
**Vasopressor use, Yes vs. No**	1.38 (1.23 to 1.54)	<0.0001
**Supervision**		
CRNA	1.06 (0.87 to 1.29)	0.5591
Combination[2]	1.27 (1.01 to 1.6)	0.04
Resident	1.32 (1.09 to 1.6)	0.0052
Solo	Ref	
**Off-hours Cases (Starting between 5PM and 6:30AM), Yes vs. No**	1.27 (1.05 to 1.54)	0.0153
**Year of Surgery**		
2015	0.94 (0.81 to 1.1)	0.4258
2016	0.83 (0.72 to 0.97)	0.0177
2017	0.91 (0.78 to 1.06)	0.2272
2018	1.07 (0.90 to 1.27)	0.4192
2014	Ref	
**Emergency case, Yes vs. No**	2.50 (2.07 to 3.01)	<0.0001

[1] Others: American Indian or Alaska Native, Asian, Native Hawaiian or Other Pacific Islander. [2] Combination: Cases involving 2 of the following: solo anesthesiologist, CRNA with anesthesiologist, resident with anesthesiologist

© 2021 Bardia A et al. *JAMA Network Open.*

eTable 3. Baseline Demographic and Clinical Characteristics Stratified by Antibiotic Usage With Respect to Dosing of Antibiotics per the IDSA Guidelines in the Study Population.

Variable	Overall (N =414851)	Guideline adherent antibiotic dosing *(N = 343835)*	Guideline non-adherent antibiotic dosing *(N = 71016)*	p-Value
**Age,** Mean (SD)	57.5 (15.7)	57.2 (15.7)	58.8 (15.9)	<0.001
**Female, n (%)**	214,960 (51.8%)	176,482 (82.1%)	38,478 (17.9%)	<0.001
**BMI,** Mean (SD)	29.2 (7.0)	29.3 (6.8)	28.7 (7.6)	<0.001
**Hispanic Ethnicity**	4872 (1.2%)	3887 (79.8%)	985 (20.2%)	<0.001
**Race**				<0.001
Black	42,416 (10.2%)	34,871 (82.2%)	7545 (17.8%)	
Others[1]	18,200 (4.4%)	15,515 (85.2%)	2685 (14.8%)	
Unknown	59,015 (14.2%)	50,250 (85.1%)	8765 (14.9%)	
White	295,220 (71.2%)	243,199 (82.4%)	52,021 (17.6%)	
**Surgical Specialty**				<0.001
General Surgery	186,711 (45.0%)	157,807 (84.5%)	28,904 (15.5%)	
Gynecology	41,832 (10.1%)	36,355 (86.9%)	5477 (13.1%)	
Orthopedics	120,015 (28.9%)	101,063 (84.2%)	18,952 (15.8%)	
Urology	66,293 (16.0%)	48,610 (73.3%)	17,683 (26.7%)	
**Duration of surgery,** Median (IQR)	182.0 (125.0 – 261.0)	184.0 (128.0 – 263.0)	170.0 (108.0 – 253.0)	<0.001
**ASA class**				<0.001
1	24,736 (6.0%)	20,932 (84.6%)	3804 (15.4%)	
2	180,336 (43.5%)	152,220 (84.4%)	28,116 (15.6%)	
3	193,926 (46.7%)	158,016 (81.5%)	35,910 (18.5%)	
4	15,514 (3.7%)	12,380 (79.8%)	3134 (20.2%)	
5	339 (0.1%)	287 (84.7%)	52 (15.3%)	
**Blood products given,** n (%)	13,547 (3.3%)	11,437 (84.4%)	2110 (15.6%)	<0.001
**Cases with Vasopressor infusion use,** n (%)	74,094 (17.9%)	65,325 (88.2%)	8769 (11.8%)	<0.001
**Supervision**				<0.001
CRNA	240,433 (58.0%)	192,231 (80.0%)	48,202 (20.0%)	
Combination[2]	28,833 (7.0%)	23,614 (81.9%)	5219 (18.1%)	
Resident	105,243 (25.4%)	92,869 (88.2%)	12,374 (11.8%)	
Solo	40,342 (9.7%)	35,121 (87.1%)	5221 (12.9%)	
**Off-hours Cases[3],** n(%)	16,212 (3.9%)	13,607 (83.9%)	2605 (16.1%)	<0.001
**Year of Surgery**				<0.001
2014	63,053 (15.2%)	45,748 (72.6%)	17,305 (27.4%)	
2015	86,761 (20.9%)	68,548 (79.0%)	18,213 (21.0%)	
2016	100,325 (24.2%)	84,835 (84.6%)	15,490 (15.4%)	
2017	117,377 (28.3%)	102,969 (87.7%)	14,408 (12.3%)	
2018	47,335 (11.4%)	41,735 (88.2%)	5600 (11.8%)	
**Emergency Case**	12,950 (3.1%)	10,905 (84.2%)	2045 (15.8%)	<0.001

© 2021 Bardia A et al. *JAMA Network Open.*

[1] Others: American Indian or Alaska Native, Asian, Native Hawaiian or Other Pacific Islander. [2] Combination: Cases involving 2 of the following: solo anesthesiologist, CRNA with anesthesiologist, resident with anesthesiologist. [3] Cases starting between 5:00PM and 6:30AM.

© 2021 Bardia A et al. *JAMA Network Open.*

eTable 4. Multivariable Analysis Evaluating the Association of Demographic and Perioperative Factors Associated With Guideline Non-adherent Antibiotic Usage With Respect to Dosing of Antibiotics.
(Non-adherent yes vs no)

Variable	Odds Ratio (95% CI)	p-Value
Age	1.01 (1.003 to 1.016)	0.0038
Gender, Male vs. Female	0.68 (0.66 to 0.69)	<0.0001
BMI	0.982 (0.981 to 0.984)	<0.0001
Ethnicity, Hispanic vs. Non-Hispanic	1.03 (0.95 to 1.11)	0.5194
Race		*<0.0001*
Black	0.99 (0.96 to 1.02)	0.5423
Others[1]	1.07 (1.02 to 1.12)	0.0043
Unknown	1.08 (1.04 to 1.11)	<0.0001
White	Ref	
Surgical Specialty		*<0.0001*
Gynecology	0.71 (0.69 to 0.74)	<0.0001
Orthopedics	1.15 (1.12 to 1.17)	<0.0001
Urology	2.12 (2.07 to 2.18)	<0.0001
General Surgery	Ref	
Duration of surgery	0.987 (0.986 to 0.988)	<0.0001
ASA class		*<0.0001*
2	0.89 (0.86 to 0.93)	<0.0001
3	0.97 (0.93 to 1.02)	0.2411
4	1.21 (1.14 to 1.29)	<0.0001
5	1.01 (0.72 to 1.39)	0.976
1	Ref	
Blood products given, Yes vs. No	1.00 (0.95 to 1.06)	0.9974
Vasopressor use, Yes vs. No	1.03 (1.004 to 1.06)	0.0258
Supervision		*<0.0001*
CRNA	0.93 (0.90 to 0.96)	0.0001
Combination[2]	1.03 (0.98 to 1.09)	0.1845
Resident	0.84 (0.80 to 0.87)	<0.0001
Solo	Ref	
Off-hours Cases (Starting between 5PM and 6:30AM), Yes vs. No	0.91 (0.87 to 0.96)	0.0002
Year of Surgery		*<0.0001*
2015	0.53 (0.52 to 0.55)	<0.0001
2016	0.41 (0.40 to 0.43)	<0.0001
2017	0.35 (0.34 to 0.36)	<0.0001
2018	0.31 (0.30 to 0.32)	<0.0001
2014	Ref	
Emergency case, Yes vs. No	1.00 (0.95 to 1.06)	0.9547

[1] Others: American Indian or Alaska Native, Asian, Native Hawaiian or Other Pacific Islander. [2] Combination: Cases involving 2 of the following: solo anesthesiologist, CRNA with anesthesiologist, resident with anesthesiologist

© 2021 Bardia A et al. *JAMA Network Open.*

eTable 5. Baseline Demographic and Clinical Characteristics stratified by Antibiotic Usage With Respect to Choice of Antibiotics per the IDSA Guidelines in the Study Population.

Variable	Overall (N = 414851)	Guideline adherent choice of antibiotic (N = 333338)	Guideline non-adherent choice of antibiotic (N = 81513)	p-Value
**Age,** Mean (SD)	57.5 (15.7)	57.8 (15.8)	56.3 (15.7)	<0.001
**Female,** n(%)	214,960 (51.8%)	176,177 (82.0%)	38,783 (18.0%)	<0.001
**BMI,** Mean (SD)	29.2 (7.0)	29.3 (6.9)	29.1 (7.1)	<0.001
**Hispanic Ethnicity**	4872 (1.2%)	3770 (77.4%)	1102 (22.6%)	<0.001
**Race**				<0.001
Black	42,416 (10.2%)	34,535 (81.4%)	7881 (18.6%)	
Others[1]	18,200 (4.4%)	14,981 (82.3%)	3219 (17.7%)	
Unknown	59,015 (14.2%)	48,938 (82.9%)	10,077 (17.1%)	
White	295,220 (71.2%)	234,884 (79.6%)	60,336 (20.4%)	
**Surgical Specialty**				<0.001
General Surgery	186,711 (45.0%)	125,122 (67.0%)	61,589 (33.0%)	
Gynecology	41,832 (10.1%)	37,209 (88.9%)	4623 (11.1%)	
Orthopedics	120,015 (28.9%)	117,795 (98.2%)	2220 (1.8%)	
Urology	66,293 (16.0%)	53,212 (80.3%)	13,081 (19.7%)	
**Duration of surgery** Median (IQR)	182.0 (125.0 – 261.0)	187.0 (130.0 – 263.0)	160.0 (107.0 – 249.0)	<0.001
**ASA class**				<0.001
1	24,736 (6.0%)	19,483 (78.8%)	5253 (21.2%)	
2	180,336 (43.5%)	146,631 (81.3%)	33,705 (18.7%)	
3	193,926 (46.7%)	154,929 (79.9%)	38,997 (20.1%)	
4	15,514 (3.7%)	12,070 (77.8%)	3444 (22.2%)	
5	339 (0.1%)	225 (66.4%)	114 (33.6%)	
**Cases with blood products given,** n (%)	13,547 (3.3%)	10,949 (80.8%)	2598 (19.2%)	0.16
**Cases with Vasopressor infusion use,** n (%)	74,094 (17.9%)	64,320 (86.8%)	9774 (13.2%)	<0.001
**Supervision**				<0.001
CRNA	240,433 (58.0%)	189,683 (78.9%)	50,750 (21.1%)	
Combination[2]	28,833 (7.0%)	23,391 (81.1%)	5442 (18.9%)	
Resident	105,243 (25.4%)	87,873 (83.5%)	17,370 (16.5%)	
Solo	40,342 (9.7%)	32,391 (80.3%)	7951 (19.7%)	
**Off-hours Cases[3],** n(%)	16,212 (3.9%)	11,920 (73.5%)	4292 (26.5%)	<0.001
**Year of Surgery**				<0.001
2014	63,053 (15.2%)	48,193 (76.4%)	14,860 (23.6%)	
2015	86,761 (20.9%)	70,113 (80.8%)	16,648 (19.2%)	
2016	100,325 (24.2%)	80,039 (79.8%)	20,286 (20.2%)	
2017	117,377 (28.3%)	95,776 (81.6%)	21,601 (18.4%)	
2018	47,335 (11.4%)	39,217 (82.8%)	8118 (17.2%)	
**Emergency case**	12,950 (3.1%)	9167 (70.8%)	3783 (29.2%)	<0.001

© 2021 Bardia A et al. *JAMA Network Open.*

[1] Others: American Indian or Alaska Native, Asian, Native Hawaiian or Other Pacific Islander. [2] Combination: Cases involving 2 of the following: solo anesthesiologist, CRNA with anesthesiologist, resident with anesthesiologist. [3] Cases starting between 5:00PM and 6:30AM.

© 2021 Bardia A et al. *JAMA Network Open.*

eTable 6. Multivariable Analysis Evaluating the Association of Demographic and Perioperative Factors Associated With Guideline Nonadherent Antibiotic Usage With Respect to the Choice of Antibiotics for a Surgery.
(Non-adherent yes vs. no)

Variable	Odds Ratio (95% CI)	p-Value
Age	1.005 (0.999 to 1.011)	0.1189
Gender, Male vs. Female	1.23 (1.21 to 1.25)	<0.0001
BMI	1.004 (1.003 to 1.006)	<0.0001
Ethnicity, Hispanic vs. Non-Hispanic	1.07 (0.99 to 1.15)	0.0917
Race		*<0.0001*
Black	0.88 (0.86 to 0.91)	<0.0001
Others[1]	0.85 (0.81 to 0.89)	<0.0001
Unknown	0.96 (0.93 to 0.98)	0.0031
White	Ref	
Surgical Specialty		*<0.0001*
Gynecology	0.265 (0.257 to 0.275)	<0.0001
Orthopedics	0.037 (0.035 to 0.038)	<0.0001
Urology	0.465 (0.454 to 0.476)	<0.0001
General Surgery	Ref	
Duration of surgery	0.9937 (0.993 to 0.9945)	<0.0001
ASA class		*<0.0001*
2	0.89 (0.86 to 0.93)	<0.0001
3	0.84 (0.81 to 0.87)	<0.0001
4	0.76 (0.72 to 0.81)	<0.0001
5	0.70 (0.55 to 0.89)	0.0043
1	Ref	
Blood products given, Yes vs. No	1.40 (1.33 to 1.48)	<0.0001
Vasopressor use, Yes vs. No	0.80 (0.78 to 0.83)	<0.0001
Supervision		*<0.0001*
CRNA	1.15 (1.12 to 1.19)	<0.0001
Combination[2]	1.03 (0.98 to 1.08)	0.1994
Resident	0.87 (0.84 to 0.90)	<0.0001
Solo	Ref	
Off-hours Cases (Starting between 5PM and 6:30AM), Yes vs. No	1.16 (1.11 to 1.21)	<0.0001
Year of Surgery		*<0.0001*
2015	0.83 (0.81 to 0.85)	<0.0001
2016	0.85 (0.83 to 0.88)	<0.0001
2017	0.87 (0.85 to 0.90)	<0.0001
2018	0.88 (0.85 to 0.91)	<0.0001
2014	Ref	
Emergency case, Yes vs. No	1.50 (1.43 to 1.58)	<0.0001

[1] Others: American Indian or Alaska Native, Asian, Native Hawaiian or Other Pacific Islander. [2] Combination: Cases involving 2 of the following: solo anesthesiologist, CRNA with anesthesiologist, resident with anesthesiologist

© 2021 Bardia A et al. *JAMA Network Open.*

eTable 7. Baseline Demographic and Clinical Characteristics Stratified by Antibiotic Usage With Respect to Redosing of Antibiotics per the IDSA Guidelines in the Study Population.

Variable	Overall[1] (N = 68776)	Guideline adherent antibiotic usage (N = 50334)	Guideline non-adherent antibiotic usage (N = 18442)	p-Value
**Age,** Mean (SD)	57.4 (14.7)	57.1 (14.8)	58.2 (14.4)	<0.001
**Female,** n (%)	32,862 (47.8%)	23,887 (72.7%)	8975 (27.3%)	0.005
**BMI,** Mean (SD)	28.4 (24.7 – 33.2)	28.4 (24.7 – 33.2)	28.3 (24.6 – 33.1)	0.11
**Hispanic Ethnicity**	781 (1.1%)	557 (71.3%)	224 (28.7%)	0.24
**Race**				0.024
Black	6243 (9.1%)	4559 (73.0%)	1684 (27.0%)	
Others[2]	3360 (4.9%)	2535 (75.4%)	825 (24.6%)	
Unknown	9592 (13.9%)	7030 (73.3%)	2562 (26.7%)	
White	49,581 (72.1%)	36,210 (73.0%)	13,371 (27.0%)	
**Surgical Specialty**				<0.001
General Surgery	28,613 (41.6%)	19,602 (68.5%)	9011 (31.5%)	
Gynecology	8729 (12.7%)	6027 (69.0%)	2702 (31.0%)	
Orthopedics	17,526 (25.5%)	14,974 (85.4%)	2552 (14.6%)	
Urology	13,908 (20.2%)	9731 (70.0%)	4177 (30.0%)	
**Duration of surgery,** Median (IQR)	340.0 (282.0 – 421.0)	331.0 (277.0 – 403.0)	371.0 (298.0 – 474.0)	<0.001
**ASA class**				<0.001
1	2267 (3.3%)	1873 (82.6%)	394 (17.4%)	
2	26,914 (39.1%)	20,074 (74.6%)	6840 (25.4%)	
3	36,676 (53.3%)	26,176 (71.4%)	10,500 (28.6%)	
4	2879 (4.2%)	2181 (75.8%)	698 (24.2%)	
5	40 (0.1%)	30 (75.0%)	10 (25.0%)	
**Cases with blood products given,** n (%)	6794 (9.9%)	4660 (68.6%)	2134 (31.4%)	<0.001
**Cases with Vasopressor infusion use,** n (%)	22,698 (33.0%)	17,050 (75.1%)	5648 (24.9%)	<0.001
**Supervision**				<0.001
CRNA	40,129 (58.3%)	28,916 (72.1%)	11,213 (27.9%)	
Combination[3]	7946 (11.6%)	5776 (72.7%)	2170 (27.3%)	
Resident	16,830 (24.5%)	13,001 (77.2%)	3829 (22.8%)	
Solo	3871 (5.6%)	2641 (68.2%)	1230 (31.8%)	
**Off-hours Cases[4],** n(%)	1513 (2.2%)	1088 (71.9%)	425 (28.1%)	0.26
**Year of Surgery**				<0.001
2014	11,862 (17.2%)	8998 (75.9%)	2864 (24.1%)	
2015	14,055 (20.4%)	10,406 (74.0%)	3649 (26.0%)	
2016	17,291 (25.1%)	12,529 (72.5%)	4762 (27.5%)	
2017	18,282 (26.6%)	13,123 (71.8%)	5159 (28.2%)	
2018	7286 (10.6%)	5278 (72.4%)	2008 (27.6%)	
**Emergency case,** n(%)	1732 (2.5%)	1354 (78.2%)	378 (21.8%)	<0.001

© 2021 Bardia A et al. *JAMA Network Open.*

[1] Only cases that qualified for redosing were included in the redosing analysis.
[2] Others: American Indian or Alaska Native, Asian, Native Hawaiian or Other Pacific Islander. [3] Combination: Cases involving 2 of the following: solo anesthesiologist, CRNA with anesthesiologist, resident with anesthesiologist. [4] Cases starting between 5:00PM and 6:30AM.

© 2021 Bardia A et al. *JAMA Network Open.*

eTable 8. Multivariable Regression Analysis Evaluating the Association of Demographic and Perioperative Factors Associated With Guideline Nonadherent Antibiotic Usage With Respect to Redosing of Antibiotics During a Surgery. (Non-adherent yes vs. no)

Variable	Odds Ratio (95% CI)	p-Value
Age	1.022 (1.007 to 1.037)	0.0037
Gender, Female vs. Male	0.97 (0.92 to 1.01)	0.1291
BMI	0.994 (0.991 to 0.997)	0.0001
Ethnicity, Hispanic vs. Non-Hispanic	1.06 (0.89 to 1.25)	0.5064
Race		*0.5614*
Black	0.98 (0.91 to 1.05)	0.5605
Others[1]	1.06 (0.96 to 1.17)	0.2422
Unknown	1.02 (0.95 to 1.09)	0.5871
White	Ref	
Surgical Specialty		*<0.0001*
Gynecology	1.091 (1.023 to 1.164)	0.0081
Orthopedics	0.411 (0.388 to 0.436)	<0.0001
Urology	0.952 (0.901 to 1.006)	0.0788
General Surgery	Ref	
Duration of surgery	1.045 (1.043 to 1.047)	<0.0001
ASA class		*0.0019*
2	1.19 (1.04 to 1.35)	0.0093
3	1.27 (1.11 to 1.45)	0.0005
4	1.3 (1.1 to 1.53)	0.0024
5	1.61 (0.7 to 3.7)	0.2642
1	Ref	
Blood products given, Yes vs. No	1.06 (0.99 to 1.13)	0.122
Vasopressor use, Yes vs. No	0.96 (0.912 to 1.01)	0.1211
Supervision		*<0.0001*
CRNA	1.09 (0.997 to 1.19)	0.0594
Combination[2]	1.17 (1.06 to 1.30)	0.0025
Resident	1.25 (1.14 to 1.37)	<0.0001
Solo	Ref	
Off-hours Cases (Starting between 5PM and 6:30AM), Yes vs. No	1.22 (1.07 to 1.40)	0.0034
Year of Surgery		*<0.0001*
2015	1.37 (1.28 to 1.47)	<0.0001
2016	1.18 (1.10 to 1.26)	<0.0001
2017	1.11 (1.04 to 1.19)	0.0021
2018	1.11 (1.02 to 1.20)	0.0181
2014	Ref	
Emergency case, Yes vs. No	1.43 (1.23 to 1.66)	<0.0001

[1] Others: American Indian or Alaska Native, Asian, Native Hawaiian or Other Pacific Islander. [2] Combination: Cases involving 2 of the following: solo anesthesiologist, CRNA with anesthesiologist, resident with anesthesiologist

© 2021 Bardia A et al. *JAMA Network Open.*

eAppendix 1. Post Hoc Analysis Evaluating Association of SSIs After Colon Surgeries and Abdominal Hysterectomy with Antibiotic Adherence at a Hospital Level

Under the terms of consortium data use agreements, we were not permitted to investigate hospital level data in any way that would lead to identification of specific institutions within the dataset. Thus, to seek a signal between hospital level adherence and SSI rates, we pursued the following preliminary post hoc analysis.

We referred to the publicly available hospital compare database which reports SSIs after colon surgeries and abdominal hysterectomy. Using a system in which investigators were blinded to hospital identifiers, hospital compare SSI data were linked by the MPOG administrators who divided each MPOG hospital into one of three tertiles based on the SSI rates for the participating hospitals for each year. The association of antibiotic adherence rates with the SSI tertiles was then assessed. In this preliminary post hoc analysis, mixed-effects ordinal logistic regression analysis was run to assess the association between adherence rate and the tertile of hospital performance, in which each hospital was treated as a random intercept effect. To account for the longitudinal nature (2014-2018) of the data, yearly adherence rate and year of surgery were included as fixed effects. Separate models were fitted for colon surgery and abdominal hysterectomy. Only hospitals that contributed 2 or more years' records were included, which were N=20 for colon surgery and N=20 abdominal hysterectomy, respectively.

There was no statistically significant association found between hospital performance tertile and adherence rate in colon surgery group (odds ratio = 0.965, 95%

© 2021 Bardia A et al. *JAMA Network Open.*

CI: 0.894-1.042, p = 0.360). The association was also not statistically significant in the abdominal hysterectomy group (odds ratio = 0.954, 95% CI: 0.839-1.084, p = 0.459). The above analysis has several limitations. It is a post hoc analysis and given the limited sample size(N=20), may be heavily underpowered. It is a preliminary to approach the question of potential associations among IDSA metrics and SSI rates which does not include accounting for case-mix or numerous other important confounders. To formally study this association, patient level tracking of outcomes will be necessary.

© 2021 Bardia A et al. *JAMA Network Open.*

eAppendix 2. Current Procedure Terminology (CPT) Codes by Surgical Specialty

Gen. Surgery

14301 14302 15220 15240 15271 15272 15273 15275 15277 15732 15734 15736 15738 15830 19020 19110 19120 19125 19260 19300 19301 19302 19303 19304 19305 19306 19307 19355 19366 20102 20103 20999 21501 21600 21740 21811 24900 25900 25909 26910 26951 27025 27027 27057 27602 27880 27882 27886 27899 31785 32225 32480 32482 32505 32608 32652 32653 32658 32662 32663 32665 32666 32669 32672 34101 34201 34800 34803 34825 34900 35301 35302 35351 35556 35601 35646 35656 35661 35741 36478 37215 37224 37226 37607 37765 37766 38100 38120 38129 38542 38562 38564 38572 38589 38720 38724 38740 38745 38747 39540 39541 42415 42420 42815 43101 43107 43112 43117 43118 43121 43122 43123 43124 43279 43280 43281 43282 43289 43310 43325 43330 43332 43333 43334 43336 43340 43361 43415 43501 43520 43610 43611 43620 43621 43622 43631 43632 43633 43634 43640 43644 43645 43659 43770 43771 43772 43773 43774 43775 43800 43820 43832 43840 43842 43843 43845 43846 43848 43860 43870 43886 43888 44005 44010 44020 44021 44025 44050 44055 44110 44120 44125 44130 44139 44140 44141 44143 44144 44145 44146 44147 44150 44151 44155 44156 44157 44158 44160 44180 44187 44188 44202 44203 44204 44205 44206 44207 44208 44210 44211 44212 44213 44227 44238 44310 44312 44314 44316 44320 44340 44345 44346 44602 44603 44604 44605 44615 44620 44625 44626 44640 44650 44660 44661 44680 44799 44800 44820 44850 44899 44950 44955 44970 44979 45005 45020 45108 45110 45111 45112 45113 45114 45116 45119 45120 45121 45123 45126 45130 45136 45150 45160 45395 45397 45400 45402 45505 45540 45541 45550 45560 45562 45563 45820 46700 46710 46712 46750 46753 46761 46940 46947 47010 47015 47120 47122 47125 47130 47370 47379 47380 47381 47399 47400 47490 47562 47563 47564 47600 47605 47610 47612 47620 47711 47715 47760 47780 47785 48105 48120 48140 48145 48146 48148 48150 48152 48153 48154 48155 48160 48510 48520 48540 48548 48999 49000 49010 49062 49215 49250 49255 49321 49322 49323 49324 49325 49329 49402 49421 49422 49426 49505 49507 49520 49521 49525 49540 49550 49553 49555 49557 49560 49561 49565 49566 49568 49570 49572 49580 49582 49585 49587 49590 49600 49650 49651 49652 49653 49654 49655 49656 49657 49659 49900 49905 49999 60200 60210 60220 60225 60240 60252 60254 60260 60271 60500 60502 60505 60520 60522 60540 60545 60650 60699

© 2021 Bardia A et al. *JAMA Network Open.*

Gynecology

49203 49204 49205 56405 56440 56620 56630 56631 56632 56740 56810 57065 57106 57120 57135 57200 57240 57250 57260 57265 57267 57280 57282 57283 57288 57295 57296 57300 57308 57320 57425 58140 58145 58146 58150 58152 58180 58200 58210 58240 58260 58262 58263 58267 58270 58275 58290 58291 58292 58541 58542 58543 58544 58545 58546 58548 58550 58552 58553 58554 58570 58571 58572 58573 58575 58578 58700 58720 58740 58750 58940 58943 58950 58951 58952 58953 58954 58956 59151

© 2021 Bardia A et al. *JAMA Network Open.*

Orthopedics

22100 22101 22102 22110 22114 22212 22214 22220 22224 22326 22327 22524 22532 22551 22554 22556 22558 22585 22586 22590 22595 22600 22610 22612 22614 22630 22633 22800 22802 22804 22808 22810 22830 22840 22842 22843 22845 22846 22849 22850 22852 22855 22856 22857 22858 22867 23156 23210 23332 23395 23410 23470 23472 23515 23550 24075 24076 24077 24105 24358 24363 24516 24545 24685 25076 25077 25078 25115 25130 25332 25447 25575 25607 26117 26540 26615 27033 27047 27048 27049 27062 27080 27086 27130 27132 27134 27137 27138 27226 27228 27236 27244 27245 27258 27269 27282 27295 27327 27328 27329 27372 27380 27385 27425 27442 27445 27446 27447 27472 27486 27487 27488 27506 27511 27513 27514 27535 27536 27580 27615 27618 27619 27630 27638 27645 27654 27656 27702 27709 27724 27734 27759 27792 27814 27822 27823 27829 28445 28805 29806 29819 29822 29823 29824 29826 29827 29828 29873 29876 29877 29879 29880 29881 29884 29915 63001 63003 63005 63015 63017 63020 63030 63040 63042 63043 63045 63046 63047 63048 63050 63055 63056 63075 63081 63085 63090 63182 63185 63190 63191 63266 63267 63276 63277 63281 63301 63302 64722

© 2021 Bardia A et al. *JAMA Network Open.*

Urology

38570 38571 38760 38765 38770 38780 50205 50220 50225 50230 50234 50236 50240 50280 50541 50542 50543 50544 50545 50546 50548 50549 50650 50715 50727 50728 50760 50770 50780 50785 50820 50825 50830 50845 50947 50949 51040 51045 51050 51065 51525 51550 51555 51565 51570 51575 51590 51595 51596 51597 51800 51841 51960 51999 52234 52235 52240 52320 52341 52344 52345 52354 52400 52500 52601 52630 52648 53010 53210 53215 53230 53260 53400 53410 53425 53430 53440 53442 53444 53445 53446 53447 53449 53500 53899 54111 54125 54304 54360 54440 54520 54530 54640 54660 54680 54840 54900 55040 55041 55060 55150 55175 55180 55530 55801 55810 55812 55821 55831 55840 55842 55845 55866 55873 55876 55899 57270

© 2021 Bardia A et al. *JAMA Network Open.*
